# In_2_S_3_ Quantum Dots: Preparation, Properties and Optoelectronic Application

**DOI:** 10.1186/s11671-019-2992-0

**Published:** 2019-05-14

**Authors:** Rujie Li, Libin Tang, Qing Zhao, Thuc Hue Ly, Kar Seng Teng, Yao Li, Yanbo Hu, Chang Shu, Shu Ping Lau

**Affiliations:** 1 School of Physics, Beijing Institute of Technology, Beijing, 100081 China; 2 Kunming Institute of Physics, Kunming, 650223 Yunnan Province China; 3 Department of Chemistry, City University of Hong Kong, Kowloon Tong, Hong Kong; 4 College of Engineering, Swansea University, Bay Campus, Fabian Way, Swansea, SA1 8EN UK; 5 School of Materials Science and Engineering, Yunnan University, Kunming, 650091 China; 6 Department of Applied Physics, The Hong Kong Polytechnic University, Hung Hom, Kowloon, Hong Kong

**Keywords:** In_2_S_3_ QDs, Preparation, Properties, Optoelectronic application

## Abstract

Low-dimensional semiconductors exhibit remarkable performances in many device applications because of their unique physical, electrical, and optical properties. In this paper, we report a novel and facile method to synthesize In_2_S_3_ quantum dots (QDs) at atmospheric pressure and room temperature conditions. This involves the reaction of sodium sulfide with indium chloride and using sodium dodecyl sulfate (SDS) as a surfactant to produce In_2_S_3_ QDs with excellent crystal quality. The properties of the as-prepared In_2_S_3_ QDs were investigated and photodetectors based on the QDs were also fabricated to study the use of the material in optoelectronic applications. The results show that the detectivity of the device stabilizes at ~ 10^13^ Jones at room temperature under 365 nm ultraviolet light irradiation at reverse bias voltage.

## Background

Graphene-like two-dimensional nanomaterials are of great scientific and technological interests [1, 2]. Currently, there has been growing research interests in developing low-dimensional materials that exhibit unique photoelectric properties [3] and quantum dots (QDs) have gained much attraction [4]. Indium sulfide (In_2_S_3_) QDs, which belong to the group III–VI semiconductor materials [5], have many unique optoelectrical, thermal, and mechanical properties, which are suitable for numerous potential applications. For example, sulfide nanomaterials have experienced rapid development for use in solar cells [6], photodetectors [7, 8], biological imaging [9], and photocatalytic degradation [10]. There are various ways of preparing sulfide QDs, and they can be divided into two main categories, namely, ‘top-down’ and ‘bottom-up’ [11].

However, commonly used bottom-up methods, such as hydrothermal [12], template[13, 14], and microwave methods [15], have many limitations that restrict the widespread application of sulfide QDs [16]. To ensure the successful application of sulfide QDs, it is of paramount importance to develop low-cost, facile preparation method that can produce stable, reliable, and high-quality QDs material [17]. In this article, a novel preparation method that allows synthesis of In_2_S_3_ QDs at atmospheric temperature conditions has been developed by using indium chloride and sodium sulfide as indium and sulfur source respectively. The physical and photoelectric properties of the as-prepared In_2_S_3_ QDs were investigated using multiple characterization techniques.

Photoelectric device based on the In_2_S_3_ QDs were fabricated, and results show the detectivity of the device stabilizes at 10^13^ Jones under 365 nm UV irradiation at room temperature, which demonstrates In_2_S_3_ QDs have great potential applications in photodetectors. Compared with other growth methods, the reported approach is mild, facile, environmentally friendly, rapid, and cheap. Therefore, it is suitable for low-cost large-scale production of the device that also yields excellent performances. This work demonstrates a low-cost, effective fabrication technique for future application of sulfide QDs in the field of photoelectric detection.

## Methods

### Materials

Sodium sulfide (Na_2_S·9H_2_O) was purchased from Tianjin Wind Ship Chemical Testing Technology Co. Ltd., Tianjin China. Indium chloride (InCl_3_·4H_2_O) was obtained from Shanghai Aladdin Biochemical Technology Co. Ltd Shanghai, China. Sodium dodecyl sulfate was purchased from Sinopharm Chemical Reagent Co. Ltd., Shanghai, China. Dialysis bag (USA spectrum lab’s regenerated cellulose membrane, *M*_*w*_ = 300) was purchased from Shanghai Yibai Economic and Trade Co. Ltd. All of the materials above were purchased commercially and used without further purification.

### In_2_S_3_ QDs Fabrication

**Fig. 1:**
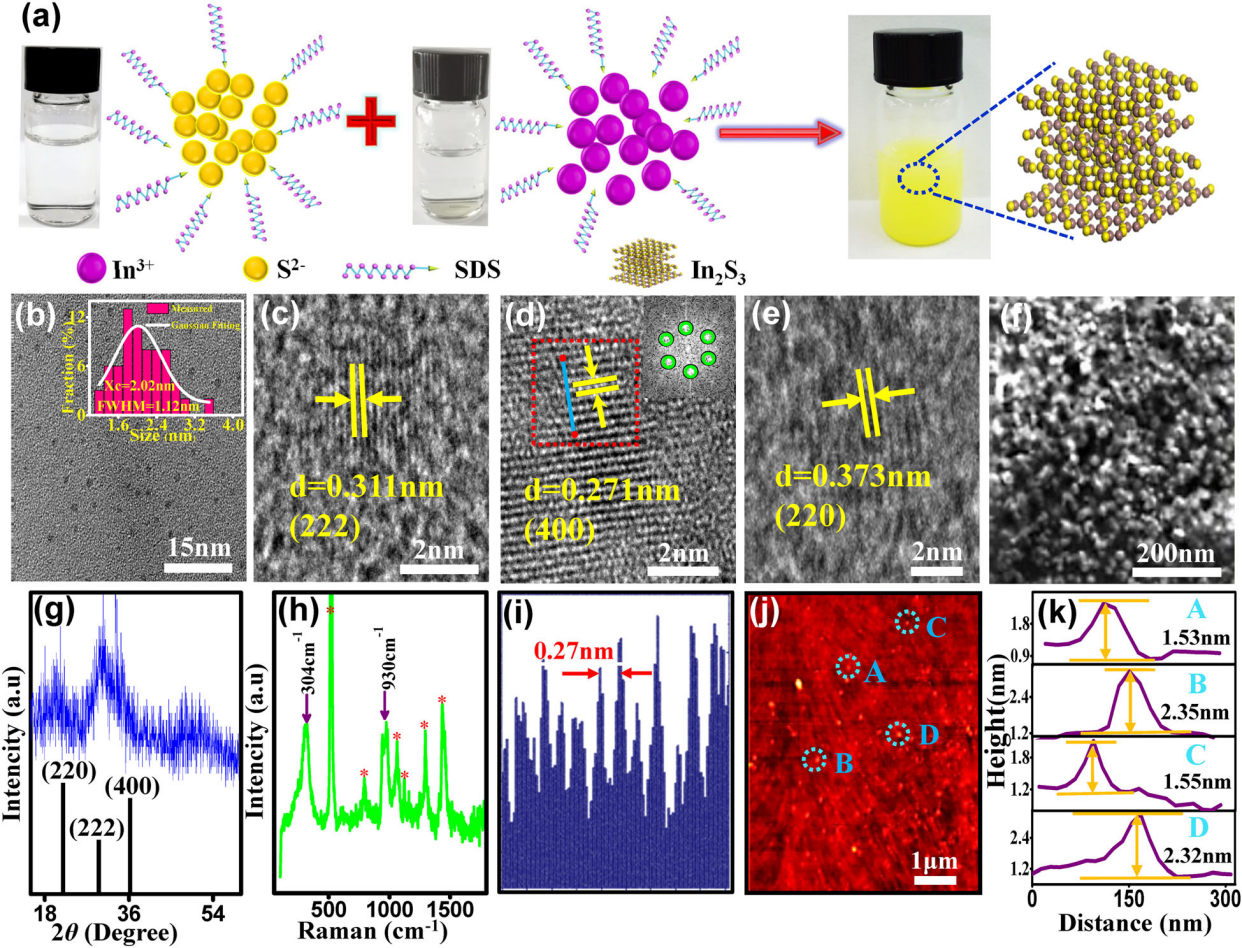
**a** The schematic illustration of the preparation of In_2_S_3_ QDs. **b** TEM image and size distribution (inset) the white line is the Gaussian fitting curve. **c**–**e** HRTEM images, inset of FFT image of a selected red area. **f** The SEM image. **g** XRD spectrum. **h** Raman spectrum. **i** The line profile of the diffraction fringes in (**d**). **j** The AFM image. **k** The height analysis of randomly selected In_2_S_3_ QDs labeled as A, B, C, and D in **j**

In_2_S_3_ QDs were prepared using the fabrication process as shown in Fig. 1a. Na_2_S (0.1 mol/L) and InCl_3_ (0.1 mol/L) were first dissolved in deionized water. The same volume of Na_2_S and SDS(CMC 0.008 mol/L)solutions were mixed using magnetic stirrer for 20 min at 1500 rpm. A mixture of InCl_3_ and SDS was prepared in the same way. The addition of SDS is to obtain a monodispersed, passivated QDs under a controlled synthesis process. The Na_2_S mixture was then added to the InCl_3_ mixture solution in a beaker to initiate the chemical reaction, which resulted in yellowish products after 10 min. Deionized water was added to the reacted solution and then followed by centrifugation at 3000 rpm for 5 min. The products were washed three times and purified using dialysis bag. The prepared In_2_S_3_ QDs were collected in the dialysis bag.

### Characterization

Transmission electron microscope (TEM) images were obtained with a JEM-2100 high-resolution trans-mission microscope operating at 200 kV. The surface morphology and phase image of photovoltaic devices were determined by scanning electron microscope (SEM, FEI Quanta 200) and AFM (atomic force microscope, SPA-400), respectively. XRD analysis was investigated using a Rigaku D/Max-RA X-ray diffractometer with Cu Ka radiation. Raman spectrum was recorded at ambient temperature on a Renishaw in via Raman microscope with an argon-ion laser at an excitation wavelength of 514.5 nm. Optical properties were characterized by UV-vis, UV-vis-NIR (UV-3600), and fluorescence (Hitachi F-7000) spectrometers. Functional groups on the surface of the In_2_S_3_ QDs were verified by XPS (X-ray photoelectron spectroscopy) (PHI Versa Probe II) using 72 W, mono Al Ka radiation. *J-V* and *C-V* were measured using Keithley 2400 source meter and semiconductor device analyzer (Keysight B1500A), respectively.

## Results and Discussion

### Structure and Morphology Studies

TEM images of the In_2_S_3_ QDs are shown in Fig. 1b–e. It can be seen that In_2_S_3_ QDs are evenly distributed and exhibit spheroid morphology. Its particle size distribution follows the Gaussian distribution with size ranging from 1 to 3 nm and FWHM of 1.12 nm. The particle has an average size of 2.02 nm. Figure 1c–e are HRTEM images of the In_2_S_3_ QDs showing its lattice fringes for *d* = 0.271 nm, 0.311 nm, and 0.373 nm, corresponding to the cubic crystal system of 400, 222, and 220 lattice planes respectively [18]. Figure 1i shows a longitudinal profile of the lattice fringes shown in Fig. 1d. The fast Fourier transform (FFT) pattern of the selected region (red dotted square) is shown in Fig. 1d insert, which reveals six bright spots from the 400 plane diffraction, indicating the crystalline structure of the hexagonal system. The scanning electron microscopy (SEM) image of the as-prepared In_2_S_3_ QDs is shown in Fig. 1f. As shown, the In_2_S_3_ QDs agglomerated to form a relatively compact structure in order to reduce its surface energy. X-ray diffraction (XRD) planes at 400, 222, and 220 of the In_2_S_3_ QDs are shown in Fig. 1g and the calculated particle size using the Sheer formula is in good agreement with the measured size from the 400 plane of HRTEM image. Figure 1h shows Raman spectrum of the In_2_S_3_ QDs with typical peaks at 304 cm^−1^ and 930 cm^−1^ [19]. Atomic force microscopy (AFM) was performed on four randomly selected In_2_S_3_ QDs, marked as A, B, C, and D as shown in Fig. 1j, with measured heights of 1.53 nm, 2.35 nm, 1.35 nm, and 2.32 nm (shown in Fig. 1k), respectively. The average height of 1.94 nm from the AFM measurement is very close to that obtained from the TEM.

The estimated band gap of In_2_S_3_ QDs is 3.50 eV, which is larger than its bulk value of 2.3 eV, due to the quantum effect. The band gap was calculated using the Brus equation:

1 $$ {E}_{np}\approx {E}_{g(0)}+\frac{{\overline{h}}^2{\pi}^2}{2{R}^2}\left(\frac{1}{{m_e}^{\ast }}+\frac{1}{{m_h}^{\ast }}\right)-\frac{1.8{e}^2}{4\pi \varepsilon R} $$

where *E*_*np*_ is the bandgap of the QDs, *E*_*g*_ is the band gap of bulk In_2_S_3_ (2.3 eV), $\overline{h}$ =h/2π is the reduced Planck constant, *e* is the electron charge, *m*_e_* is the effective mass of electron, *m*_h_^*^ is the effective mass of hole, *m*_e_^*^= *m*_h_^*^(0.25 × 10^−28^g), *R* is the radius of the particle and *ε* is the dielectric constant (*ε =* 11).

**Fig. 2:**
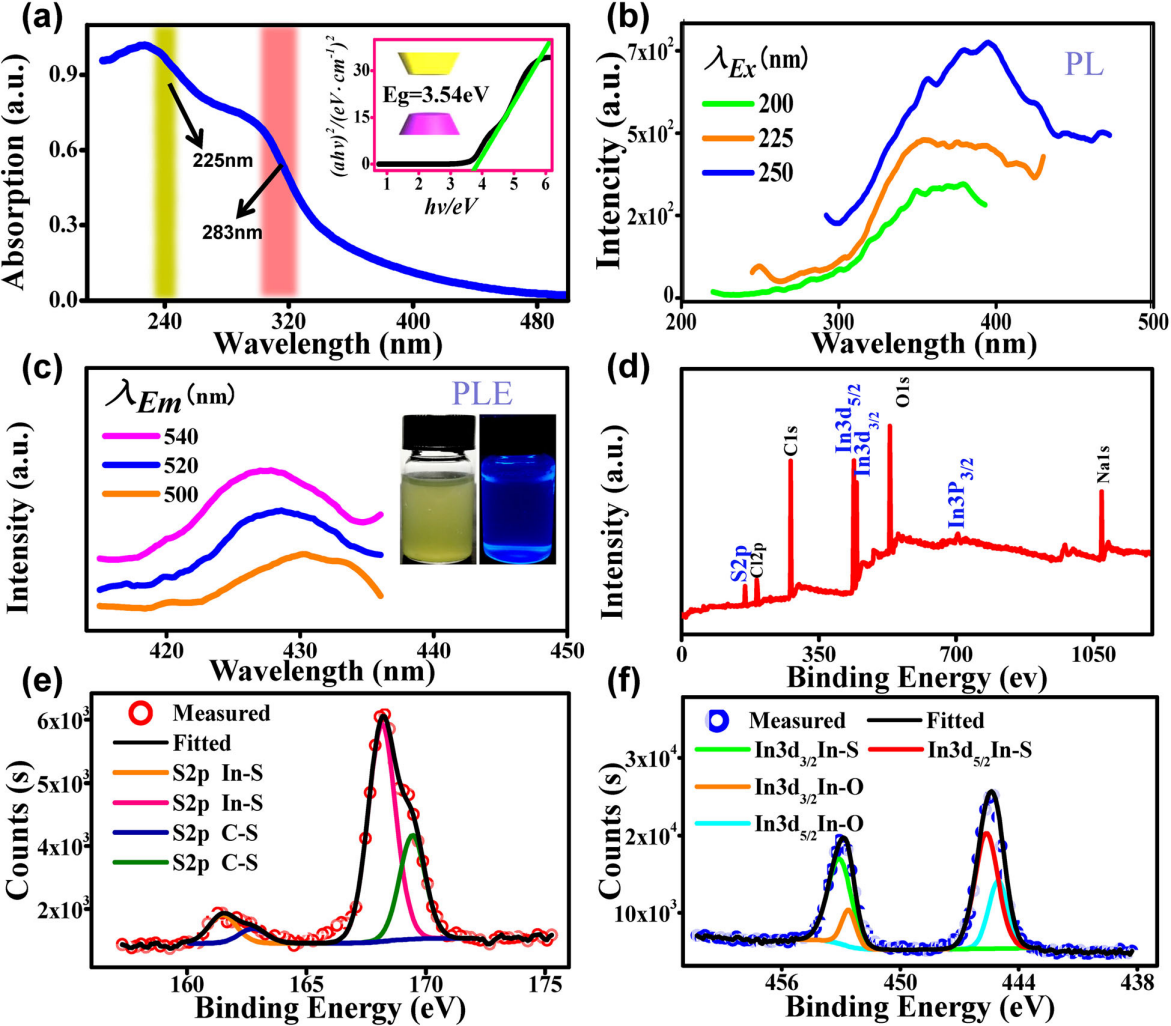
**a** UV-vis absorption spectra of In_2_S_3_ QDs aqueous solution. Inset: an estimation of band gap energy (*E*_*g*_). **b** PL emission spectra. **c** PL excitation (PLE) spectra, inset: luminescence image under visible and 365 nm light source. **d** The XPS full-scan spectrum. **e** XPS S2p spectrum. **f** XPS In3d_3/2_ and In3d_5/2_ spectrum

Figure 2a shows ultraviolet-visible (UV-vis) absorption spectra of the In_2_S_3_ QDs. There are two characteristic peaks of absorption located at 225 nm and 283 nm [20]. Since In_2_S_3_ is a direct bandgap material, its optical band gap can be expressed by the following equation:

2 $$ \alpha hv=A{\left( hv- Eg\right)}^{1/ 2} $$

where *α* is the absorption coefficient, *A* is a constant, *hv* is the photo energy, and *Eg* is the band gap energy.

The band gap energy of the QDs can be estimated from the curve of (*αhv*)^2^ vs. photo energy (*hv*). The estimated *E*_*g*_ of 3.54 eV, as shown in the inset of Fig. 2a, is very close to the calculated value using the Brus equation (*E*_*np*_=3.50 eV). Photoluminescence (PL) and photoluminescence excitation (PLE) [21] studies were performed to investigate the optical properties of the In_2_S_3_ QDs. It can be seen from Fig. 2b that there is an emission peak at a wavelength between 300 and 450 nm, and the strongest peak intensity is centered at ~ 390 nm under the excitation of *E*_x_ = 250 nm. PLE spectra in Fig. 2c show that wavelengths of the characteristic excitation peaks are shorter than the receiving wavelengths (500–540 nm). The broadening of energy gap of In_2_S_3_ QDs compared to its bulk material may also be demonstrated by PL and PLE results. The fluorescence of the In_2_S_3_ QDs under visible light and 365 nm UV light are shown in Fig. 2c insert. This demonstrates that the In_2_S_3_ QDs possess good UV fluorescence properties. X-ray photoelectron spectroscopy (XPS) was also performed to study the chemical bonds of the In_2_S_3_ QDs. Figure 2d shows the XPS full scan spectrum, which consists of S2p at 162.5 eV, In3d_5/2_ at 444.5 eV, and In3d_3/2_ at 452.5 eV. Besides, there are residual Cl, Na, O, and C from the surfactant and reactant. Core level peaks of S2p and In3d are shown in Fig. 2e, f respectively. The deconvoluted peaks reveal the bonding states of S2p (In-S, C-S), In3d_5/2_. (In-S, In-O), and In3d_3/2_ (In-S, In-O).

**Fig. 3:**
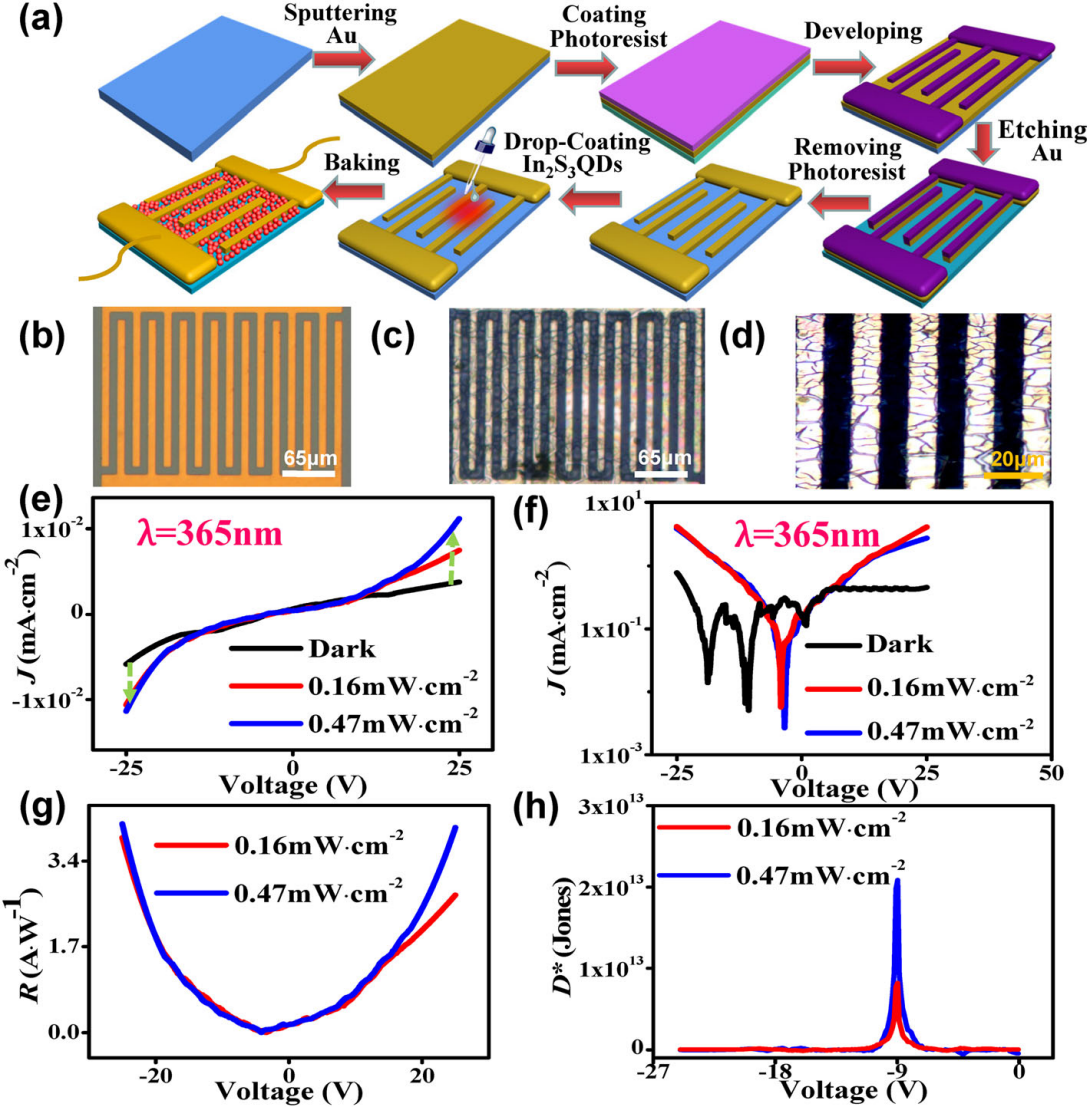
**a** Schematic diagram illustrating the fabrication process of the In_2_S_3_ QDs UV photovoltaic detector. **b** Electrode without QD. **c**–**d** Optical microscopic images of the In_2_S_3_ QDs photodetector at different magnifications. **e**–**h** Performance of the In_2_S_3_ QDs detector. **e** *J-V* curves. **f** Log *(J)-V* curves. **g** *R* (responsivity)*-V* curves. **h** D*

As the In_2_S_3_ QDs demonstrated excellent ultraviolet absorption properties, UV photodetector based on the In_2_S_3_ QDs was fabricated and investigated. The preparation process is illustrated in Fig. 3a.

The specification of the Au interdigitated electrodes is similar to that reported by Tang. et al. [22], consisting of electrodes with a thickness of 400 nm, a length of 120 μm, and width and spacing of 10 μm. Figure 3b shows an optical image of empty electrodes. Fig. 3c, d shows the optical microscopic images showing the spacing of the electrodes filled with the In_2_S_3_ QDs, which acted as a photosensitive layer. The measured current density against voltage (*J*-*V*) and log (*J*-*V*) curves of the device in dark condition, irradiated by 0.16 mW cm^−2^ and 0.47 mW cm^−2^ power density of 365 nm UV light are shown in Fig. 3e, f respectively. An increase in the current density is observed when the irradiated power density increases, hence demonstrating the characteristics of a rectifier. The responsivity (*R*) and detectivity (D*) of the photodetector are calculated using the following equations:

3 $$ R={J}_{\mathrm{ph}}/{P}_{\mathrm{opt}} $$

4 $$ D\ast =\frac{R}{\sqrt{2q/ jd}} $$

where *J*_ph_ is the photocurrent density, *P*_opt_ is the photo power density, *q* is the absolute electron charge (1.6 × 10^−19^ coulombs), and *J*_*d*_ is the dark current density [23]. From Fig. 3g, the maximum value of *R* is 4.13 A W^−1^, which is significantly larger than that of graphene and many other two-dimensional nanomaterial devices [24, 25] and is seen to increase with an increase in the reverse bias voltage. As shown in Fig. 3h, the D* is stabilized at around 10^13^ Jones.

**Fig. 4:**
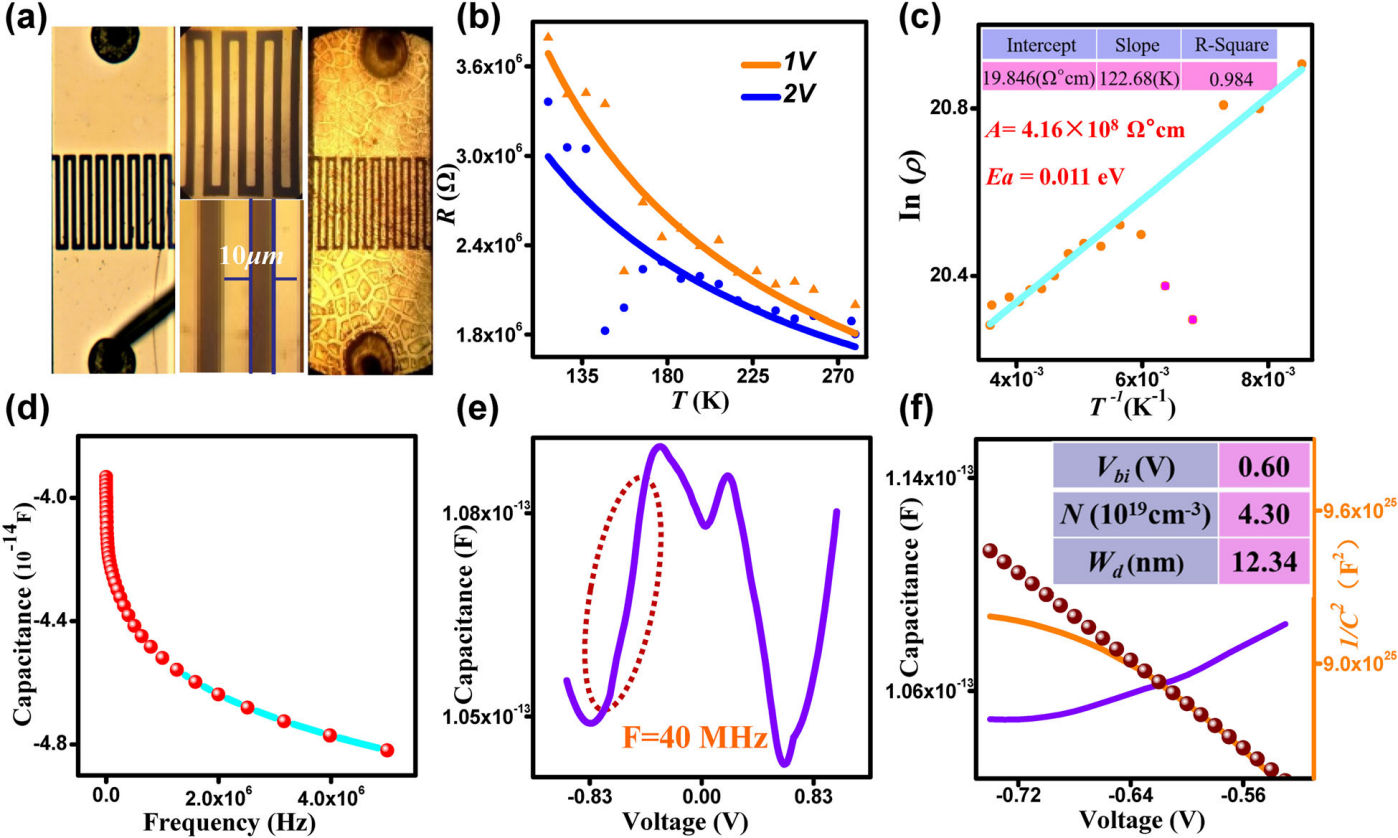
**a** Photodetector with In_2_S_3_ QDs as an active layer. **b** Plot of R-T at 1 V and 2 V. **c** Plot of *ln (ρ)-1/T-*based devices at 1 V. **d** *C-F* curves measured at room temperature. **e** The *C-V* curves (40 MHz)-based photodetector in the dark condition. **f** Variation of the capacitance with applied voltages and plots of *1/C*^*2*^ vs*. V* of the device

The optical images of empty electrodes and those filled with In_2_S_3_ QDs are shown in Fig. 4a. The plot of *R*-*T* measured from the In_2_S_3_ QDs-based photodetector at a voltage of 1 V and 2 V is shown in Fig. 4b. It shows that an increase in temperature has led to a decrease in the resistance; however, it does not exhibit a simple linear relationship. In order to understand the electrical properties of the In_2_S_3_ QDs, the ln*(ρ)*-1/*T* of the device was attained and the results are shown in Fig. 4c. By using the two model equations [26]:

5 $$ \rho =R\frac{\left(N-1\right)\kern0em wd}{l} $$

6 $$ \mathrm{In}\ \left(\rho \right)\kern0.5em =\kern0.5em \mathrm{In}\kern0.5em (A)\kern0.5em +\kern0.5em {E}_a/\kern0.5em \left({k}_b\cdot T\right) $$

where *N* is the number of interdigitated electrodes, *w* is the overlapping length, *l* is the spacing, and *d* is the thickness of the film [27]. Using a simple linear regression, the calculated thermal activation energy (*E*_*a*_) is 0.011 eV and the finger-leading factors (*A*) is 4.16 × 10^8^ Ω°cm. The thermal activation energy of In_2_S_3_ QDs could be reduced as long as the obtained energy is sufficient for the carriers to participate in conduction, which can result in lower resistivity and higher conductivity.

Generally, *C*-*V* measurements can provide many important information on the nature of the semiconductor interface and charge transport. Fig. 4d shows that capacitance decreases with increasing frequency and the decrease in capacitance is significant at low frequencies. This is due to the interface states, which respond to the alternating current signal, and the presence of the interface states would suppress the AC signal at high frequency, hence resulting in a weakened trend or a constant capacitance. Figure 4e shows the *C-V* curves of the In_2_S_3_ QDs-based photodetector at room temperature with a frequency of 40 MHz*.* The *C-V* relationship under a bias can be expressed as [28]

7 $$ {C}^{-2}=\frac{2\left({V}_{bi}-V\right)}{q{\varepsilon}_0{\varepsilon}_r{NS}^2} $$

where *V*_bi_ is the built-in potential at zero bias, *ε*_*0*_ is the permittivity of vacuum, ε_*r*_ is the relative permittivity of a material, *N* is the carrier concentration in the depletion layer and *S* is the photosensitive area (3.3 mm^2^). The x-intercept is *V*_bi_ = 0.6 V, and the carrier concentration *N* can be calculated from the slope of the linear section of *1/C*^2^ vs. *V* plot [29]: $N=\frac{-2}{q{\varepsilon}_0{\varepsilon}_r{A}^2}{\left[\frac{\partial \left({C}^{-2}\right)}{\partial V}\right]}^{-1}$, and the calculated *N*=4.3 × 10^19^ cm^−3^. The depletion width (*W*_*d*_) is between the electrode and the In_2_S_3_ QDs layer, expressed as ${W}_d={\left[\frac{2{\varepsilon}_0{\varepsilon}_r\left({V}_{bi}-V\right)}{qN}\right]}^{1/2}$, the calculated *W*_*d*_ *=* 12.34 nm. These physical parameters are shown in Fig. 4f. It is evident that the *V*_bi_ and *W*_*d*_ are the same as similar QDs devices (such as the graphene quantum dots) [30], but the *N* is larger by an order of magnitude at zero bias. This explains the excellent performances of the device as compared to other QDs device [31].

## Conclusions

A novel and facile preparation method to produce high crystal quality In_2_S_3_ QDs was developed. The structural, optical, electrical, and photovoltaic properties of the In_2_S_3_ QDs have been studied. In the dark field condition, the activation energy (*E*_*a*_), finger-leading factor (*A*), built-in potential (*V*_bi_), and depletion layer width (*W*_*d*_) of the UV photodetector based on In_2_S_3_ QDs were obtained. In_2_S_3_ QDs were used as the sole photoactive material in the fabricated photodetector that exhibits the highest detectivity (D*) of 2 × 10^13^ Jones at room temperature under 365 nm UV light illumination without preamplifier. This method is ideal in developing high performance, large array of In_2_S_3_ QDs-based UV photoelectric detector at very low cost.

## Abbreviations

AFM: Atomic force microscope
CMC: Critical micelle concentration
FFT: Fast Fourier transform
FWHM: Full width at half maximum
HRTEM: High-resolution transmission electron microscope
PL: Photoluminescence
PLE: Photoluminescence excitation
QDs: Quantum dots
SDS: Sodium dodecyl sulfate
SEM: Scanning electron microscope
TEM: Transmission electron microscope
XPS: X-ray photoelectron spectroscopy
XRD: X-ray diffractometer
